# Cost-effectiveness of the Namaste care family program for nursing home residents with advanced dementia in comparison with usual care: a cluster-randomized controlled trial

**DOI:** 10.1186/s12913-020-05570-2

**Published:** 2020-09-04

**Authors:** Mohamed El Alili, Hanneke J. A. Smaling, Karlijn J. Joling, Wilco P. Achterberg, Anneke L. Francke, Judith E. Bosmans, Jenny T. van der Steen

**Affiliations:** 1 Department of Health Sciences, Faculty of Science, Vrije Universiteit Amsterdam, Amsterdam Public Health Research Institute, Amsterdam, The Netherlands; 2 Department of Public and Occupational Health, Amsterdam UMC, Vrije Universiteit Amsterdam, Amsterdam Public Health Research Institute, Amsterdam, The Netherlands; 3 Department of Public Health and Primary Care, Leiden University Medical Center, Leiden, The Netherlands; 4 Department of General Practice and Elderly Care Medicine, Amsterdam UMC, Vrije Universiteit Amsterdam, Amsterdam Public Health Research Institute, Amsterdam, The Netherlands; 5 Netherlands Institute for Health Services Research (NIVEL), Utrecht, The Netherlands; 6 Expertise Center Palliative Care, Amsterdam UMC, Vrije Universiteit Amsterdam, Amsterdam, The Netherlands; 7 Department of Primary and Community Care, Radboud university medical center, Nijmegen, The Netherlands

**Keywords:** Dementia, Quality of life, Namaste care, Cluster randomized controlled trial, Family caregiving, Person-centered care, Palliative care, Nursing home, Cost-benefit analysis

## Abstract

**Background:**

Dementia is a progressive disease that decreases quality of life of persons with dementia and is associated with high societal costs. The burden of caring for persons with dementia also decreases the quality of life of family caregivers. The objective of this study was to assess the societal cost-effectiveness of Namaste Care Family program in comparison with usual care in nursing home residents with advanced dementia.

**Methods:**

Nursing homes were randomized to either Namaste Care Family program or usual care. Outcome measures of the cluster-randomized trial in 231 residents included Quality of Life in Late-Stage Dementia (QUALID) and the Gain in Alzheimer Care Instrument (GAIN) for family caregivers over 12 months of follow-up. Health states were measured using the EQ-5D-3L questionnaire which were translated into utilities. QALYs were calculated by multiplying the amount of time a participant spent in a specific health state with the utility score associated with that health state. Healthcare utilization costs were estimated using standard unit costs, while intervention costs were estimated using a bottom-up approach. Missing cost and effect data were imputed using multiple imputation. Bootstrapped multilevel models were used after multiple imputation. Cost-effectiveness acceptability curves were estimated.

**Results:**

The Namaste Care Family program was more effective than usual care in terms of QUALID (− 0.062, 95%CI: − 0.40 to 0.28), QALY (0.0017, 95%CI: − 0.059 to 0.063) and GAIN (0.075, 95%CI: − 0.20 to 0.35). Total societal costs were lower for the Namaste Care Family program as compared to usual care (− 552 €, 95%CI: − 2920 to 1903). However, these differences were not statistically significant. The probability of cost-effectiveness at a ceiling ratio of 0 €/unit of effect extra was 0.70 for the QUALID, QALY and GAIN.

**Conclusions:**

The Namaste Care Family program is dominant over usual care and, thus, cost-effective, although statistical uncertainty was considerable.

**Trial registration:**

Netherlands Trial Register (http://www.trialregister.nl/trialreg/index.asp, identifier: NL5570, date of registration: 2016/03/23).

## Background

Worldwide, 50 million people live with dementia. Total estimated costs caused by dementia were $948 billion in 2016 with Europe and North-America experiencing the highest economic burden [1]. Because of the progressive nature of dementia, quality of life in people with dementia and their family members is severely affected [2–5]. There are several psychosocial interventions available for people with dementia, but there are not many interventions that specifically target people with advanced dementia [6]. Moreover, psychosocial interventions are generally not part of end-of-life care. However, there is evidence that appropriate end-of-life care in advanced dementia effectively addresses patients’ and families’ concerns and needs [7].

People with advanced dementia are often admitted to long-term care facilities. Although care in such facilities is primarily taken over by professionals, the burden for family caregivers (i.e. family, relatives, friends) often remains high [8]. In addition, family caregivers sometimes find it difficult to sustain a meaningful connection with people with advanced dementia with decreased communication skills. Family caregivers may be frustrated, especially if possibilities for verbal communication are limited due to for example aphasia. As a result, visits might be experienced as stressful [9]. Existing psychosocial interventions for people with dementia living in long-term care facilities usually do not include family caregivers. Moreover, effects of such interventions on family caregiver experiences are generally not evaluated.

Namaste Care is a psychosocial intervention for people with advanced dementia that entails person-centered care for people with advanced dementia involving nursing staff and volunteers [10]. The primary aim of the program is to improve the quality of life of people with advanced dementia by focusing on a meaningful connection with them, which might result in positive effects in people with dementia and their family caregivers. Studies reported that Namaste Care successfully improves the quality of life of people with advanced dementia and their family caregivers without extra healthcare costs [11–13]. Despite the fact that these studies have estimated costs and effects associated with Namaste Care, no full economic evaluation has been performed yet. Furthermore, Namaste Care reduced behavioral symptoms of dementia as well as the use of psychotropic medication [14–16]. However, the aforementioned studies primarily focused on the qualitative (i.e. subjective) aspect of Namaste Care. So far, no randomized controlled trials have been performed that assessed the (cost-) effectiveness of Namaste Care. Therefore, the aim was to assess the cost-effectiveness of an adapted version of Namaste Care, the Namaste Care Family program, in comparison with usual care among people with advanced dementia and their family caregivers.

## Methods

### Study design

The economic evaluation was conducted from a societal perspective alongside a cluster-randomized controlled trial that compared the Namaste Care Family program with usual care over a time horizon of 12 months. The protocol was evaluated by the Medical Ethics Review Committee of the VU University Medical Center in Amsterdam, the Netherlands (protocol number: 2016.399) and is registered in the Netherlands Trial Register (http://www.trialregister.nl/trialreg/index.asp, identifier: NL5570). The committee declared that no further formal review was needed, since the study was declared exempt from the scope of the Medical Research Involving Human Subjects Act. Detailed information on the study design and the Namaste Care Family program trial is provided elsewhere [17]. Funding sources were not involved in the design and execution of the study or writing of the study results.

### Recruitment and setting

Nineteen Dutch nursing homes with a psychogeriatric ward were recruited. Dutch nursing homes offer long-term care, some in a domestic-small-scale living style environment. It includes 24-h functional support and (medical and nursing) care for people that are in need of assistance with activities of daily living.

### Randomization and sample size

Participating nursing homes were matched on several characteristics that potentially affect the outcomes. These characteristics were collected by means of a questionnaire sent out to the managers of the nursing homes and included questions on the number of beds, whether the nursing home offered small-scale living arrangements for persons with dementia yes or no, whether it was situated in a rural or urban area, and the number of offered psychosocial programs such as “Snoozelen”. Furthermore, the religious orientation of the nursing home was also used as a matching criterion when it was felt it affected end-of-life decision-making care practice. Possible matches were judged by three researchers (HJAS, KJJ and JTvdS). Subsequently, matched pairs of nursing homes were randomized by an independent statistician. Due to the nature of the intervention, the group allocation could not be masked. Sample size calculations indicated that with 192 participants, power sufficed to detect a relevant difference in participants’ quality of life [17].

### Recruitment of participants

Within each nursing home, nursing staff was asked to indicate potentially eligible residents. Residents were eligible if they had advanced dementia and were unable to participate in regular activity programs, or if they had moderate dementia with behavioral symptoms of dementia such as agitation, aberrant motor behavior, aggression or apathy. If the nursing staff was of the opinion that the resident or family caregiver could benefit from the program, for example because the resident was responsive to touch, the resident was considered eligible. Further, eligible residents had family caregivers with sufficient proficiency in Dutch who were willing and able to fill in questionnaires. Written informed consent was provided by family caregivers. No financial incentive to participate was provided.

Ultimately, nineteen nursing homes consented to participate and were allocated to the Namaste Care Family program (*n* = 10) or usual care (*n* = 9).

### Intervention

Namaste Care is a multidimensional care program with psychosocial, sensory and spiritual components that incorporates tailored and personalized care until death for people with advanced dementia. Experiences and outcomes of family caregivers may be improved when their involvement in the program is increased. Hence, we expanded the program by also inviting family caregivers to training sessions and involve them in Namaste Care, which is referred to as the Namaste Care Family program [10]. Ideally, this Namaste Care Family program was given 7-days-a-week in two 2-h sessions [10]. The sessions took place in a calm room with a ‘home-like’, relaxed setting, the so-called ‘Namaste-room’. In this ‘Namaste-room’, pleasant scents were used and nature sounds or soft music were played. Furthermore, attempts were made to avoid external distractions or interruptions. Each session started with personally welcoming each participant when entering the Namaste-room. Each participant was comfortably seated and screened for signs of pain. Appetizing, nutritious foods and drinks were offered frequently. The main aim was to establish a meaningful connection between participants and family caregivers. Extra personal care (e.g. massages, grooming, nail care and washing the face, hands and feet) was offered during the sessions to aid in an experience of a gentle, caring touch. Each session ended with nursing staff personally thanking each participant for attending the session. A more detailed description of the intervention can be found elsewhere [17]. In the control group, participants only received usual care, which was not restricted in any way.

### Clinical outcomes

Data were collected between May 2016 and December 2018. Primary clinical outcomes were the Quality of Life in Late-Stage Dementia (QUALID) [18], the three-level version of the EuroQol (EQ-5D-3L) [19], and the Gain in Alzheimer Care Instrument (GAIN) [20], which were assessed at baseline, and 1, 3, 6 and 12 months after baseline. The QUALID is a proxy-rated quality of life instrument for people with dementia and consists of 11 items [18, 21]. Each items has 5 response options. Summed scores range from 11 to 55 with lower scores indicating better quality of life. The QUALID has good psychometric properties in people with advanced dementia [18, 22, 23]. The EQ-5D-3L contains five dimensions (mobility, self-care, activities of daily living, pain/discomfort and depression/anxiety) with 3 answer levels (no problems to severe problems). Both the EQ-5D-3L and QUALID were filled in by nursing staff. The EQ-5D-3L health states were converted to utility scores using the Dutch tariff, where 0 is anchored to death and 1 to full health (range − 0.30 to 1, where negative utilities indicate that a health state is valued as worse than death) [19]. Using the area under the curve method, QALYs were calculated by multiplying the amount of time a participant spent in a specific health state with the utility score associated with that health state. Transitions between health states were linearly interpolated. The GAIN measures family caregivers’ gains in dementia caregiving [20]. The scale has 10 items that are scored on a Likert scale from 0 (disagree a lot) to 4 (agree a lot). Summed scores can range from 0 to 40, with higher scores indicating higher gains. The GAIN has good psychometric properties [20].

### Costs

Costs were measured from a societal perspective (secondary care costs, medication costs, family costs and Namaste Care Family program costs) using questionnaires primarily based on a Dutch standardized data collection tool for older nursing home residents, the TOPIC-MDS [24] at 1, 3, 6, and 12 months after baseline. Additional questions on the costs of the Namaste Care Family program were developed specifically for this study. Medication use was assessed using medication data from the nursing homes.

Healthcare utilization was valued using standard costs from the Dutch costing guideline [25]. Medication costs were valued using prices from the Royal Dutch Pharmacists Association [26]. Family costs included time spent with the participant, administrative tasks for the participant, travel time and distance to visit the participant, finding replacement for daily activities when visiting, and lost productivity due to family caregivers’ absenteeism from work. The shadow price of these time investments is assumed to be equal to the tariff for cleaning work. Lost productivity costs due to absenteeism from work were calculated using gender-specific income values of the Dutch population.

The Namaste Care Family program costs were estimated using a bottom-up micro-costing approach, which included costs of supplies for the intervention, any change (increase or decrease) in nursing staff time as well as hiring extra nursing staff, and family and volunteer time investments. Costs related to supplies and other investments, as well as actual costs of donated items, were collected by asking the participating nursing homes to estimate the monetary value of supplies and donations they received. Extra staff costs were estimated using their hourly wage. All costs were expressed in Euros for the year 2018 using consumer price indices [27]. Discounting was not necessary, because follow-up was restricted to 12 months.

### Statistical analysis

The cost-effectiveness analyses were conducted according to the intention-to-treat principle. Missing data were replaced using Multiple Imputation with Chained Equations (MICE) [28]. Cost and effect data were assumed to be missing at random, which means that missing observations are explained by observed variables [29]. The imputation model included outcome variables and predictor variables that either differed at baseline, were related to missing data or were associated with the outcome (see Table 2 for variables included in imputation model). To account for the skewed distribution of cost data, predictive mean matching was used in MICE [30]. The number of imputed datasets was increased until the loss of efficiency was less than 5%, resulting in 10 imputed datasets [30]. Each of the imputed datasets was analyzed separately as described below. Results from the multiple datasets were pooled using Rubin’s rules [31].

Multilevel regression models were used to estimate incremental costs and effects between the treatment groups, while accounting for the clustering of the data by allowing the intercepts to vary across clusters (i.e. random intercepts model). For costs and QALYs, a two-level structure was used where nursing homes and participants represented the first and second level, respectively. QALYs were adjusted for baseline utility. For the difference in QUALID and GAIN, an additional level accounted for repeated observations within persons (i.e. scores at different time points). The differences in QUALID and GAIN were additionally adjusted for confounders (see Table 3 for list of confounders). Incremental Cost-Effectiveness Ratios (ICERs) were calculated by dividing the incremental costs by incremental effects. Bias-corrected bootstrapping was used to estimate statistical uncertainty (2000 replications). Statistical uncertainty surrounding ICERs was illustrated by plotting the bootstrapped cost-effect pairs on a cost-effectiveness plane (CE plane). Cost-effectiveness acceptability curves (CEACs) were also estimated, which demonstrate the probability that the Namaste Care Family program is cost-effective compared to usual care for a range of different ceiling ratios (i.e. the willingness-to-pay threshold for one point effect extra) [32]. In the Netherlands, the generally used willingness-to-pay threshold for healthcare interventions ranges between 10,000 and 80,000 € per QALY gained [33]. For outcome measures such as the QUALID and GAIN, no willingness-to-pay thresholds have been determined. Analyses were performed in IBM SPSS Statistics 24® (IBM Corp., Armonk, NY, US), StataSE 14® (StataCorp LP, CollegeStation, TX, US) and MLwiN® (University of Bristol, Bristol, UK) [34] from within StataSE 14® [35]. To check the robustness of the results, four sensitivity analyses were performed. First, the economic evaluation was performed without adjustment for confounders (SA1). Second, it was performed from the healthcare perspective (SA2), which included secondary care costs, medication costs, and Namaste Care Family program costs. Third, clustering was ignored in the estimation of incremental costs and effects (SA3). Finally, the economic evaluation was performed using observed data, i.e. missing data was handled using complete-case analysis (SA4).

## Results

### Population

Table 1 presents baseline characteristics for participants in both groups. In total, 231 residents and their family caregivers participated. Two participating nursing homes randomized to the Namaste Care Family program dropped out after 3 months and 6 months, respectively. During follow-up, 60 (52%) participants in the intervention group (*n* = 116) and 63 (55%) participants in the usual care group (*n* = 115) had incomplete QALY data, mostly due to death. For total societal costs, these figures were 44 (19%) and 49 (21%), respectively, which was due to selective drop-out i.e. death of participant. Supplementary Table 1 summarizes the probability of having incomplete data for different baseline variables. The probability of having incomplete data was twice as high for participants that were born in the Netherlands as compared to participants born outside the Netherlands (odds ratio = 2.31, *P* = 0.034). The probability of having incomplete data was also twice as high for participants older than 80 years old as compared to participants younger than 80 years (e.g. for QALY the odds ratio = 2.11, *P* = 0.026). The number of family caregivers that participated in the Namaste Care Family program (*n* = 116) ranged from 33 (28%) family caregivers at one month after baseline to 15 (13%) family caregivers at the end of the trial. On average, family caregivers participated in 3.4 sessions per month.

**Table 1:** Descriptives of the Namaste Care Family group and usual care group at baseline

	Namaste Care Family program		Usual care		
	n	Mean (SD) / % (n)	n	Mean (SD) / % (n)	*P*-value^c^
**Person with dementia**					
Age (years)	110	83.3 (8.1)	113	86.0 (6.9)	.010*
Gender (% female)	116	71% (82)	114	75% (85)	.510
% born in the Netherlands	108	82% (88)	110	94% (103)	.006*
Educational level					.374
None or primary school	106	43% (45)	110	35% (39)	
(High school preparing for) technical/trade school	106	44% (47)	110	54% (59)	
High school preparing for BSc or MSc	106	4% (4)	110	6% (6)	
BSc or MSc degree	106	9% (10)	110	6% (6)	
Dementia severity^a^	115	14.7 (4.8)	112	15.3 (4.2)	.294
Utility^b^ (0–1)	115	0.46 (0.29)	112	0.44 (0.24)	.460
**Family caregivers**					
Age (years)	108	61.8 (11.1)	107	63.4 (11.4)	.297
Gender (% female)	108	75% (81)	110	68% (75)	.265
% born in the Netherlands	108	85% (92)	110	97% (107)	.002*
Educational level					.112
None or primary school	108	2% (2)	110	8% (9)	
(High school preparing for) technical/trade school	108	52% (56)	110	52% (57)	
High school preparing for BSc or MSc	108	8% (9)	110	11% (12)	
BSc or MSc degree	108	38% (41)	110	29% (32)	
Relation with person with dementia					.578
Spouse / partner	109	22% (24)	112	17% (19)	
Daughter or son (in law)	109	65% (71)	112	66% (74)	
Other	109	14% (15)	112	15% (17)	

^a^ as measured by the Bedford Alzheimer Nursing Severity-Scale ^b^ as measured by the EQ-5D-3L ^c^ Baseline differences were tested using t-tests for continuous variables and chi-square tests for categorical variables ^*^ *P* < .05, BSc Bachelor, MSc Master, SD Standard Deviation

### Clinical outcomes (QUALID, GAIN and QALY)

Differences in QUALID and GAIN scores between the Namaste Care Family program and usual care were small and statistically not significant (Table 2). In addition, the mean difference in QALYs between the groups was not statistically significant (Table 2).

**Table 2:** Multiply imputed effects and costs for the Namaste program group (*n* = 116) and usual care group (*n* = 115) after 12 months

Outcomes		Mean (SE)		Mean difference (95%CI)^c^
		Namaste Care Family program (n = 116)	Usual care (n = 115)	
Quality of Life in Late-Stage Dementia (QUALID) score (11–55)^b^	Baseline	24.19 (0.80)	22.37 (0.71)	−0.060 (− 0.42; 0.30)^a^
	T1	24.95 (0.77)	22.87 (0.74)	
	T2	24.51 (0.79)	22.46 (0.73)	
	T3	23.88 (0.89)	21.85 (0.74)	
	T4	23.40 (2.25)	22.51 (1.04)	
Gain in Alzheimer Care Instrument (GAIN) score (0–40)	Baseline	24.94 (0.80)	22.77 (0.82)	0.033 (−0.24; 0.31)^a^
	T1	24.96 (0.81)	22.60 (1.03)	
	T2	23.93 (1.02)	23.06 (0.93)	
	T3	22.09 (1.21)	23.32 (0.99)	
	T4	26.26 (1.74)	23.62 (1.37)	
Quality Adjusted Life Year (QALY) (0–1)		0.40 (0.026)	0.39 (0.022)	0.015 (−0.058; 0.088)
**Healthcare costs**				
Secondary care		251 (110)	361 (151)	−110 (− 529; 215)
Medication		5430 (777)	6432 (1047)	− 1002 (− 3971; 1268)
Total healthcare costs		5682 (802)	6793 (1065)	− 1111 (− 4071; 1246)
Donations		105 (16)	0 (0)	105 (76; 139)
Structural		211 (29)	0 (0)	211 (163; 276)
Extra staff		477 (89)	0 (0)	477 (325; 681)
Total intervention costs		793 (110)	0 (0)	793 (602; 1036)
**Non-healthcare costs**				
Family		698 (121)	691 (131)	7 (− 288; 303)
**Total societal costs**		7173 (852)	7484 (1087)	− 311 (− 3397; 2097)

^a^Overall effect over time corrected for score at baseline

^b^A lower QUALID score indicates improved health. A higher QUALID score indicates worse health

^c^Uncertainty around cost differences estimated using the non-parametric bootstrap

SE standard error, 95%CI 95% confidence interval

Multiple imputation model consisted of variables that differed at baseline, were related to missing data or were associated with the outcome: age of family caregiver, age of person with dementia, country of birth of person with dementia, country of birth of family caregiver, gender person with dementia, gender of family caregiver, relation of family caregiver with person with dementia, marital status of person with dementia, highest completed education of person with dementia and having a payed job for the family caregiver

### Costs

Mean total intervention costs amounted to 793 € per participant (Table 2). Total healthcare costs were lower in the Namaste Care Family program compared to usual care, but this difference was not significant (− 1111 €, 95%CI: − 4071 to 1246). Medication costs were the main contributor to this cost difference (− 1002 €, 95%CI: − 3971 to 1268). The difference in family costs between the Namaste Care Family program and usual care was small and not significant (7 €, 95%CI: − 288 to 303). The difference in total societal costs was 311 € in favor of the intervention group, which was not significant (95% CI: − 3397 to 2097).

### Cost-effectiveness and cost-utility

**Fig. 1:**
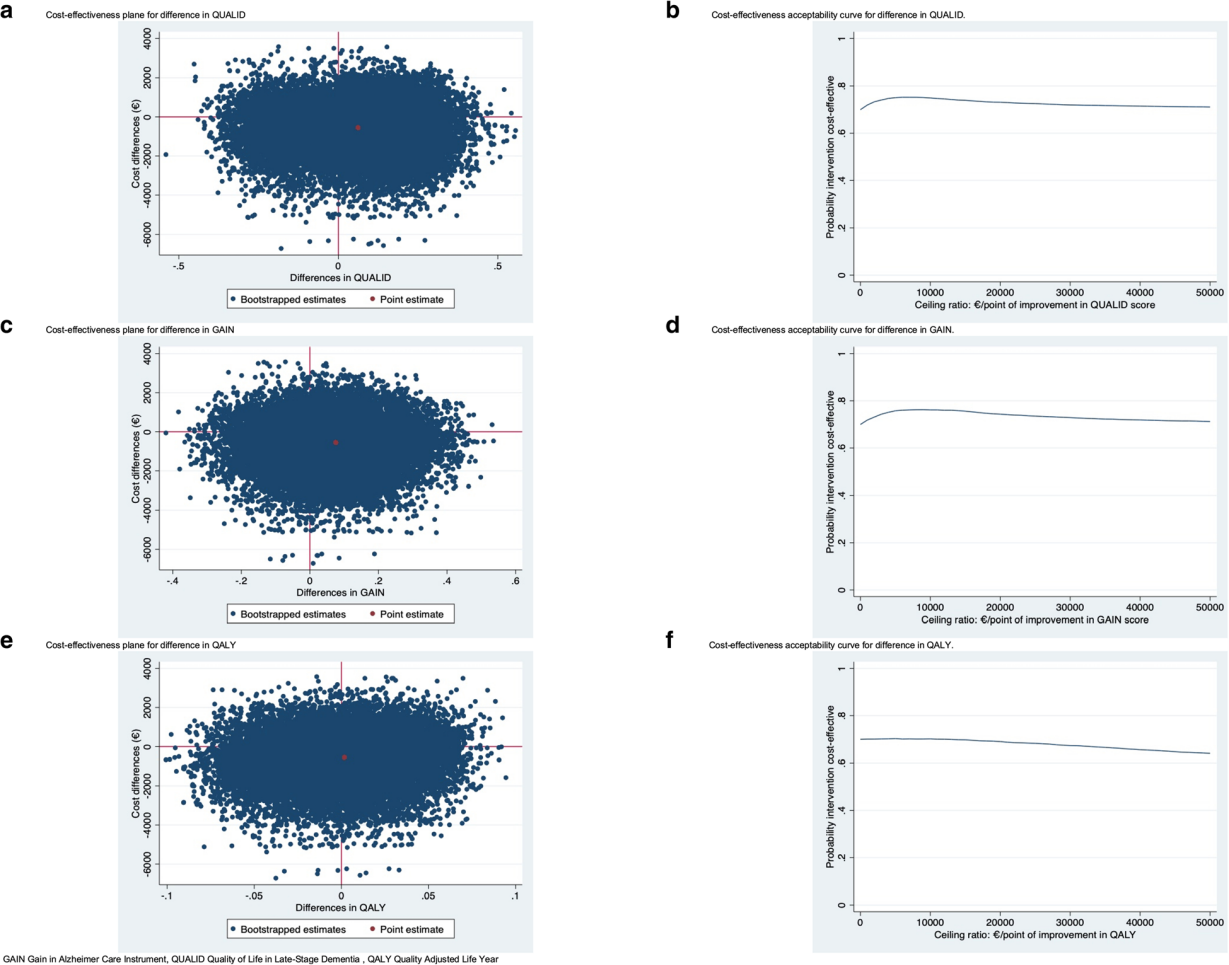
Cost-effectiveness planes and cost-effectiveness acceptability curves

For the QUALID, the ICER was 8919 (Table 3), meaning that 1 point of improvement in QUALID score is associated with a saving of 8919 € in the Namaste Care Family group compared with the usual care group. Thus, the Namaste Care Family program was dominant over usual care. At ceiling ratios of 0, 10,000 and 20,000 € per point of improvement on the QUALID, the probability that the Namaste Care Family program is cost-effective compared to usual care is 0.70, 0.75 and 0.73, respectively (Fig. 1b).

**Table 3:** Results of the cost-effectiveness analysis, cost-utility analysis and sensitivity analyses

Outcome	ΔC (95% CI) ^d^	ΔE (95% CI)	ICER	CE plane			
				NE	SE	SW	NW
**Main analysis: Societal perspective**							
Quality of Life in Late-Stage Dementia (QUALID) score (11–55)	− 552 (− 2920; 1903)	−0.062 (− 0.40; 0.28) ^a,b^	8919	21%	49%	21%	9%
Gain in Alzheimer Care Instrument (GAIN) score (0–40)	− 552 (− 2920; 1903)	0.075 (− 0.20; 0.35) ^a,c^	−7310	20%	48%%	22%	10%
QALYs (0–1)	−552 (− 2920; 1903)	0.0017 (− 0.059; 0.063)	−315,671	17%	35%	35%	13%
**SA1: Unadjusted analysis**							
Quality of Life in Late-Stage Dementia (QUALID) score (11–55)	−552 (− 2920; 1903)	− 0.060 (− 0.40; 0.28)^a^	9158	21%	49%	21%	9%
Gain in Alzheimer Care Instrument (GAIN) score (0–40)	−552 (− 2920; 1903)	0.033 (− 0.26; 0.32)^a^	−16,913	16%	37%	33%	14%
QALYs (0–1)	−552 (− 2920; 1903)	0.015 (− 0.058; 0.088)	− 36,774	20%	46%	24%	10%
**SA 2: Healthcare perspective**							
Quality of Life in Late-Stage Dementia (QUALID) score (11–55)	− 548 (− 2805; 1927)	− 0.062 (− 0.40; 0.28) ^a,b^	8861	19%	50%	21%	9%
Gain in Alzheimer Care Instrument (GAIN) score (0–40)	− 548 (− 2805; 1927)	0.075 (− 0.20; 0.35) ^a,c^	− 7263	19%	49%	23%	9%
QALYs (0–1)	− 548 (− 2805; 1927)	0.0017 (− 0.059; 0.063)	− 313,640	16%	36%	35%	13%
**SA 3: Ignore clustering of data**							
Quality of Life in Late-Stage Dementia (QUALID) score (11–55)	− 311 (− 3340; 2227)	0.15 (− 0.27; 0.47) ^a,b^	− 2065	7%	10%	49%	34%
Gain in Alzheimer Care Instrument (GAIN) score (0–40)	− 311 (− 3340; 2227)	0.18 (− 0.12; 0.48) ^a,c^	− 1714	37%	51%	7%	5%
QALYs (0–1)	− 311 (− 3340; 2227)	0.0018 (− 0.058; 0.062)	− 169,209	22%	32%	26%	20%
**SA 4: Complete-case analysis**							
Quality of Life in Late-Stage Dementia (QUALID) score (11–55)	214 (− 7863; 5630)	− 0.20 (− 0.53; 0.12) ^a,b^	− 1059	46%	45%	5%	4%
Gain in Alzheimer Care Instrument (GAIN) score (0–40)	214 (− 7863; 5630)	− 0.15 (− 0.44; 0.15) ^a,c^	− 1469	2%	13%	37%	48%
QALYs (0–1)	214 (− 7863; 5630)	0.026 (− 0.12; 0.072)	− 8118	13%	13%	37%	37%

^a^Overall effect over time corrected for score at baseline

^b^QUALID was adjusted for age person with dementia, gender of person with dementia, education of person with dementia. A lower score indicates improved health. A higher score indicates decreases health

^c^GAIN was adjusted for age family caregiver, gender family caregiver, education family caregiver, relationship between person with dementia and family caregiver

^d^ Uncertainty around cost differences estimated using the non-parametric bootstrap

CE plane cost-effectiveness plane, ICER incremental cost-effectiveness ratio, SA sensitivity analysis, 95%CI 95% confidence interval

CE-plane quadrants:

NE North-east quadrant indicates higher costs and improved health for Namaste in comparison with usual care, NW North-west quadrant indicates higher costs and worse health, SE South-east quadrant indicates lower costs and improved health, SW South-west quadrant indicates lower costs and worse health

For the GAIN, the ICER was − 7310 (Table 3), indicating that improvement of 1 point in GAIN score is associated with a saving of 7310 € in the Namaste Care Family program as compared to the usual care group. Thus, the Namaste Care Family program was dominant over usual care. The CEAC (Fig. 1d) shows that the probability that the Namaste Care Family program was cost-effective compared to usual care is 0.70, 0.76 and 0.74 when the ceiling ratio was set at 0, 10,000 and 20,000 € per point improvement on the GAIN, respectively.

The cost-utility analysis resulted in an ICER of − 315,671 (Table 3). This indicates that for each QALY that is gained, 315,671 € is saved in the Namaste Family Care program compared with the usual care group. Thus, the Namaste Care Family program was dominant over usual care. The CEAC (Fig. 1f) shows that the probability that the Namaste Care Family program was cost-effective compared to usual care is 0.70, 0.70 and 0.69 when the ceiling ratio was set at 0, 10,000 and 20,000 € per additional QALY, respectively.

### Sensitivity analysis

**Fig. 2:**
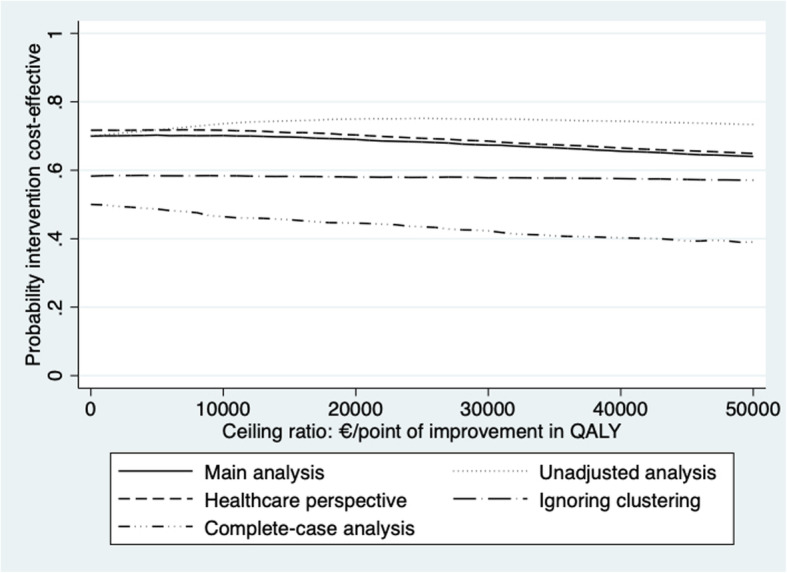
Cost-effectiveness acceptability curves of all the sensitivity analyses for QALY

Results of the unadjusted analyses (SA1) were somewhat different for the GAIN score and QALY. For GAIN, the incremental effect decreased from 0.075 to 0.033, resulting in the ICER being doubled from − 7310 to − 16,913, meaning that improvement of 1 point on the GAIN score is associated with a saving of 16,913 €. For QALY, the incremental effect increased, which resulted in a smaller ICER, i.e. -36,744. The cost-effectiveness analyses from a healthcare perspective (SA2) were comparable to the main analyses (Table 3). Ignoring clustering in the economic evaluation (SA3), resulted in a smaller cost saving of the Namaste Care Family program as compared to usual care than in the main analysis. As a consequence, the probability of cost-effectiveness at 0 € per additional unit of effect (0.58) was lower than in the main analysis (0.70). The complete-case analysis (SA4) had a large impact on the outcomes. The difference in societal costs became positive (214 €, 95%CI: − 7863 to 5630), the difference in QUALID score decreased, the difference in GAIN score became negative and the difference in QALY increased. Figure 2 graphically illustrates the sensitivity analysis in terms of the probability of cost-effectiveness for QALYs.

## Discussion

### Main findings

We found no statistically significant differences in costs and clinical outcomes between the Namaste Care Family program and usual care, although overall clinical outcomes improved and costs were lower. When considering statistical uncertainty, the probability of the Namaste Care Family program being cost-effective compared to usual care at a ceiling ratio of 0 € per additional unit of effect is 0.70. Based on these results, the Namaste Care Family program is dominant over and cost-effective compared to usual care, however, some uncertainty is present which indicates that these results should be interpreted with caution.

### Interpretation of findings and comparison with literature

Although not statistically significant, the direction of the outcomes of the cost-effectiveness analyses of the Namaste Care Family program is in line with previously published studies that showed that Namaste Care can improve quality of life nursing home residents with advanced dementia in the United Kingdom [11, 16].

In line with previous studies investigating the implementation of the Namaste Care program [11, 12], no statistically significant differences in costs between the Namaste Care Family program and usual care were found. However, a recent costing study that developed a full cost model (i.e. mapping all relevant individual cost components based on resources needed for the intervention) for Namaste Care in the United Kingdom, estimated higher staff costs for Namaste Care as compared to usual care than in the current study. In that study, staff costs were the largest contributor to total Namaste Care costs [13]. In the current economic evaluation, the intervention costs amounted to 793 €, including staff costs of 477 € which is relatively low compared to the estimated costs in the UK cost model [13]. A possible explanation is that the UK study also included capital costs (e.g. costs related to Namaste room hire in care homes), while in the current economic evaluation these type of costs were assumed to be included in the standard costs to estimate the intervention costs [25]. Also, more involvement of family caregivers may reduce costs of Namaste Care, and, thereby, increase the efficiency of the program. In the current study, the involvement of family caregivers was only modest. It is important to consider that involvement of family caregivers could increase the burden and strain on family caregivers, which adversely impacts the health of family caregivers [36, 37]. However, it can also decrease burden and provide positive experiences for family caregivers. In the current study the burden on family caregivers was not increased [38].

### Strengths and limitations

A strength of the current economic evaluation is the pragmatic design, meaning that actual practice is resembled as much as possible, which increases the generalizability of the results. Another strength relates to the societal perspective used, meaning that all relevant costs were taken into account regardless of who pays for them. This enables the identification of potential cost shifts between sectors, for example from the healthcare system to the patient and his/her family. In addition, clustering at the level of nursing homes was accounted for in the analyses. For the QUALID and GAIN, an additional level was added for the repeated observations over time. Clustering of data is frequently ignored in cost-effectiveness analyses alongside cluster-randomized controlled trials [39]. Appropriately accounting for clustering of data (main analysis) versus ignoring clustering of data (SA3) shows that ignoring clustering can affect the estimates of an economic evaluation substantially, thereby potentially changing the conclusion of an economic evaluation.

There are also a number of limitations of the current study. In residents with dementia, the use of proxies is a common approach to overcome difficulties associated with the assessment of outcomes. Some argue that quality of life is difficult to be estimated by proxies [40]. However, a number of studies [41, 42] suggest that information from proxy raters are sensitive to actual changes in quality of life, especially when the proxy is a family caregiver. A recent systematic review found no significant difference in quality of life scores between family caregivers and staff proxy ratings for nursing home residents with dementia [43]. Although the EQ-5D is the preferred instrument to estimate utility scores in economic evaluations, it may not capture all important aspects of psychosocial care for advanced dementia [44–46]. Therefore, specific dementia outcomes, i.e. the QUALID and GAIN for family caregivers of persons with dementia, were also assessed in this study [46]. Last, a considerable amount of data was missing, which was accounted for by using multiple imputation; this is considered the most appropriate method to deal with missing data in economic evaluations [47].

## Conclusion

This study showed that the Namaste Care Family program is dominant over usual care, primarily due to cost savings. Therefore, the Namaste Care Family program can be considered cost-effective compared to usual care. However, there is a considerable amount of statistical uncertainty surrounding the results. Therefore, these results should be interpreted with caution. Future research should investigate whether the results can be confirmed in different settings.

## Supplementary information

**Additional file 1 Supplementary Table 1.**

## Data Availability

The datasets used and/or analyzed during the current study are available from the corresponding author on reasonable request.

## Abbreviations

95%CI: 95% Confidence Interval
BSc: Bachelor
CE plane: Cost-Effectiveness plane
CEAC: Cost-Effectiveness Acceptability Curve
EQ-5D-3L: EuroQol
GAIN: Gain in Alzheimer Care Instrument
ICER: Incremental Cost-Effectiveness Ratio
MICE: Multiple Impuation by Chained Equations
MSc: Master
NE: North East
NW: North West
QALY: Quality Adjusted Life Year
QUALID: Quality of Life in Late-Stage Dementia
SA: Sensitivity Analysis
SD: Standard Deviation
SE: Standard Error
SE: South East
SW: South West

## References

[1] Xu J, Zhang Y, Qiu C, Cheng F (2017). Global and regional economic costs of dementia: a systematic review. Lancet.

[2] Arcand M, Monette J, Monette M, Sourial N, Fournier L, Gore B (2009). Educating nursing home staff about the progression of dementia and the comfort care option: impact on family satisfaction with end-of-life care. J Am Med Dir Assoc.

[3] Boller F, Verny M, Hugonot-Diener L, Saxton J (2002). Clinical features and assessment of severe dementia. A review 1. Eur J Neurol.

[4] Brookes RL, Herbert V, Paul S, Hannesdottir K, Markus HS, Morris RG (2014). Executive dysfunction, awareness deficits and quality of life in patients with cerebral small vessel disease: a structural equation model. Neuropsychology..

[5] Hugo J, Ganguli M (2014). Dementia and cognitive impairment: epidemiology, diagnosis, and treatment. Clin Geriatr Med.

[6] Kverno KS, Black BS, Nolan MT, Rabins PV (2009). Research on treating neuropsychiatric symptoms of advanced dementia with non-pharmacological strategies, 1998–2008: a systematic literature review. Int Psychogeriatr.

[7] Van der Steen JT, Radbruch L, Hertogh CM, de Boer ME, Hughes JC, Larkin P (2014). White paper defining optimal palliative care in older people with dementia: a Delphi study and recommendations from the European Association for Palliative Care. Palliat Med.

[8] Metzelthin SF, Verbakel E, Veenstra MY, Van Exel J, Ambergen AW, Kempen GI (2017). Positive and negative outcomes of informal caregiving at home and in institutionalised long-term care: a cross-sectional study. BMC Geriatr.

[9] Piechniczek-Buczek J, Riordan ME, Volicer L (2007). Family member perception of quality of their visits with relatives with dementia: a pilot study. J Am Med Dir Assoc.

[10] Simard J, Ladislav V (2007). The end-of-life Namaste care program for people with dementia.

[11] Stacpoole M, Hockley J, Thompsell A, Simard J, Volicer L (2017). Implementing the Namaste care program for residents with advanced dementia: exploring the perceptions of families and staff in UK care homes. Ann Palliative Med.

[12] Stacpoole M, Thompsell A. OA25 The namaste care programme can enrich quality of life for people with advanced dementia and those who care for them without additional resources: BMJ Supportive & Palliative Care. 2015;5:A8. 10.1136/bmjspcare-2015-000906.25 25960537

[13] Bray J, Brooker D, Latham I, Wray F, Baines D. Costing resource use of the Namaste care intervention UK: a novel framework for costing dementia care interventions in care homes. Int Psychogeriatr. 2019:1–10. https://www.cambridge.org/core/journals/international-psychogeriatrics/article/costing-resource-use-of-the-namaste-care-intervention-uk-a-novelframework-for-costing-dementia-care-interventions-in-care-homes/6E482849E42AF6098215B9285AF6DF4D. 10.1017/S1041610218002314 30786947

[14] Fullarton J, Volicer L (2013). Reductions of antipsychotic and hypnotic medications in Namaste care. J Am Med Dir Assoc.

[15] Simard J, Volicer L (2010). Effects of Namaste care on residents who do not benefit from usual activities. Am J Alzheimers Dis Other Demen.

[16] Stacpoole M, Hockley J, Thompsell A, Simard J, Volicer L (2015). The Namaste care programme can reduce behavioural symptoms in care home residents with advanced dementia. Int J Geriatr Psychiatry.

[17] Smaling HJA, Joling KJ, van de Ven PM, Bosmans JE, Simard J, Volicer L (2018). Effects of the Namaste care family programme on quality of life of nursing home residents with advanced dementia and on family caregiving experiences: study protocol of a cluster-randomised controlled trial. BMJ Open.

[18] Weiner MF, Martin-Cook K, Svetlik DA, Saine K, Foster B, Fontaine CS (2000). The quality of life in late-stage dementia (QUALID) scale. J Am Med Dir Assoc.

[19] Lamers LM, McDonnell J, Stalmeier PF, Krabbe PF, Busschbach JJ (2006). The Dutch tariff: results and arguments for an effective design for national EQ-5D valuation studies. Health Econ.

[20] Yap P, Luo N, Ng WY, Chionh HL, Lim J, Goh J (2010). Gain in Alzheimer care INstrument--a new scale to measure caregiving gains in dementia. Am J Geriatr Psychiatry.

[21] Schalkwijk D, Verlare LR, Muller MT, Knol DL, van der Steen JT (2009). Measuring quality of life in nursing home residents with severe dementia: psychometric properties of the QUALID scale. Tijdschr Gerontol Geriatr.

[22] Falk H, Persson LO, Wijk H (2007). A psychometric evaluation of a Swedish version of the quality of life in late-stage dementia (QUALID) scale. Int Psychogeriatr.

[23] Martin-Cook K, Hynan LS, Rice-Koch K, Svetlik DA, Weiner MF (2005). Responsiveness of the quality of life in late-stage dementia scale to psychotropic drug treatment in late-stage dementia. Dement Geriatr Cogn Disord.

[24] Lutomski JE, Baars MA, Schalk BW, Boter H, Buurman BM, den Elzen WP (2013). The development of the older persons and informal caregivers survey minimum DataSet (TOPICS-MDS): a large-scale data sharing initiative. PLoS One.

[25] Hakkaart van Roijen L, Van der Linder N, Bouwmans C, Tan S (2016). Richtlijn voor het uitvoeren van economische evaluaties in de gezondheidszorg.

[26] Z-index. G-standaard 2019 [Available from: https://www.z-index.nl/g-standaard. Accessed March 2019.

[27] Netherland S (2017). Statline consumer pricing index.

[28] van Buuren S, Groothuis-Oudshoorn K. Mice: multivariate imputation by chained equations in R. Journal of Statistical Software. 2011;45(3):1-67.

[29] Faria R, Gomes M, Epstein D, White IR (2014). A guide to handling missing data in cost-effectiveness analysis conducted within randomised controlled trials. PharmacoEconomics..

[30] White IR, Royston P, Wood AM (2011). Multiple imputation using chained equations: issues and guidance for practice. Stat Med.

[31] Rubin DB (1987). Multiple imputation for nonresponse in surveys.

[32] Fenwick E, O'Brien BJ, Briggs A (2004). Cost-effectiveness acceptability curves--facts, fallacies and frequently asked questions. Health Econ.

[33] Karpenko AW, Geenen JW, Vreman RA, Hovels A (2017). The introduction of a threshold for the Icer and the implications for reimbursement of drugs in the Dutch healthcare system. Value Health.

[34] Rabash J, Charlton C, Browne WJ, Healy M, Cameron B (2009). MLwiN version 2.36.

[35] Leckie G, Charlton C (2013). Runmlwin-a program to run the MLwiN multilevel modelling software from within stata. J Stat Softw.

[36] Galvin J (2013). The importance of family and caregiver in the care and management of people with Alzheimer's disease. Alzheimers Dement.

[37] National Academies of Sciences E, Medicine. Families caring for an aging America: National Academies Press; 2016. 27905704

[38] Smaling H, Smaling H, Joling K, Doncker S, Achterberg W, van der Steen J (2019). Perceived impact of the Namaste care family program on people with advanced dementia, nursing staff, and family caregivers: a qualitative study. J Am Med Dir Assoc.

[39] Gomes M, Grieve R, Nixon R, Edmunds WJ (2012). Statistical methods for cost-effectiveness analyses that use data from cluster randomized trials: a systematic review and checklist for critical appraisal. Med Decis Making.

[40] Arons AM, Krabbe PF, Schölzel-Dorenbos CJ, van Der Wilt GJ, Rikkert MGO (2013). Quality of life in dementia: a study on proxy bias. BMC Med Res Methodol.

[41] Bryan S, Hardyman W, Bentham P, Buckley A, Laight A (2005). Proxy completion of EQ-5D in patients with dementia. Qual Life Res.

[42] Milne D, Mulder L, Beelen H, Schofield P, Kempen G, Aranda S (2006). Patients’ self-report and family caregivers’ perception of quality of life in patients with advanced cancer: how do they compare?. Eur J Cancer Care.

[43] Robertson S, Cooper C, Hoe J, Hamilton O, Stringer A, Livingston G (2017). Proxy rated quality of life of care home residents with dementia: a systematic review. Int Psychogeriatr.

[44] Dzingina MD, McCrone P, Higginson IJ (2017). Does the EQ-5D capture the concerns measured by the palliative care outcome scale? Mapping the palliative care outcome scale onto the EQ-5D using statistical methods. Palliat Med.

[45] Wichmann AB, Adang EMM, Stalmeier PFM, Kristanti S, Van den Block L, Vernooij-Dassen MJFJ (2017). The use of quality-adjusted life years in cost-effectiveness analyses in palliative care: mapping the debate through an integrative review. Palliat Med.

[46] Sopina E, Chenoweth L, Luckett T, Agar M, Luscombe GM, Davidson PM (2019). Health-related quality of life in people with advanced dementia: a comparison of EQ-5D-5L and QUALID instruments. Qual Life Res.

[47] Burton A, Billingham LJ, Bryan S (2007). Cost-effectiveness in clinical trials: using multiple imputation to deal with incomplete cost data. Clin Trials.

