# Supplementary material for: Cost-effectiveness of the Namaste care family program for nursing home residents with advanced dementia in comparison with usual care: a cluster-randomized controlled trial
Source: BMC Health Serv Res. 2020 Sep 4;20:831. doi: 10.1186/s12913-020-05570-2 (PMC7473814; doi:10.1186/s12913-020-05570-2)
Supplement: Supplementary file 1 — Additional file 1 Supplementary Table 1. [file 12913_2020_5570_MOESM1_ESM.docx]

| **Outcome (complete vs. incomplete data)** | **Baseline variables** | **Odds ratio (OR)** | **P-value** |
| --- | --- | --- | --- |
| Quality of Life in Late-Stage Dementia (QUALID) score | Gender (men vs. woman) | 1.13 | .70 |
|  | Country of birth (Netherlands vs. other) | 1.23 | .55 |
|  | Education (Higher education vs. other) | 1.40 | .33 |
|  | Age person with dementia (older than 80 vs. younger than 80) | 2.36 | .026* |
| Gain in Alzheimer Care Instrument (GAIN) score | Gender (men vs. woman) | 1.12 | .70 |
|  | Country of birth (Netherlands vs. other) | 0.70 | .32 |
|  | Education (Higher education vs. other) | 1.50 | .26 |
|  | Age person with dementia (older than 80 vs. younger than 80) | 2.05 | .05 |
| Quality Adjusted Life Year (QALY) | Gender (men vs. woman) | 1.24 | .56 |
|  | Country of birth (Netherlands vs. other) | 1.49 | .25 |
|  | Education (Higher education vs. other) | 1.30 | .46 |
|  | Age person with dementia (older than 80 vs. younger than 80) | 2.11 | .043* |
| Total societal costs | Gender (men vs. woman) | 0.86 | .62 |
|  | Country of birth (Netherlands vs. other) | 2.31 | .034* |
|  | Education (higher education vs. other) | 0.64 | .22 |
|  | Age person with dementia (older than 80 vs. younger than 80) | 0.76 | .44 |
| An OR larger than 1 indicates higher odds of having missing data. An OR of smaller than 1 indicates lower odds of having missing data.  *P<.05 | | | |
